# Pennsylvania State Board of Veterinary Medical Examiners

**Published:** 1901-02

**Authors:** W. Horace Hoskins

**Affiliations:** Secretary


Secretary.

### PENNSYLVANIA STATE BOARD OF VETERINARY MEDICAL EXAMINERS.

The annual meeting of this Board for the examination of applicants desiring to obtain the license enabling them to practice veterinary medicine and surgery in the State of Pennsylvania was held at City Hall, Philadelphia, June 18 and 19, 1900. Eighteen applicants, representing the Veterinary Department of the University of Pennsylvania, United States College of Veterinary Surgeons, McKillip Veterinary College, Chicago Veterinary College, and Ontario Veterinary College, presented themselves before the Board, this being the second examination for two of the applicants. Licenses were granted to all but two, they having failed in some branches. Election of officers for the ensuing year resulted in the selection of Drs. James W. Sallade as President and W. Horace Hoskins as Secretary-Treasurer.

A vote of thanks was extended the Secretary-Treasurer for the work accomplished during the year after the presentation of an extended report of the work done in looking after alleged violations of the law.

THE semi-annual meeting of this Board was held at Philadelphia, Pa., December 17 and 18, 1900. Two applicants presented themselves for examination, having failed to pass the Board in June.

The action of the Board in dealing with a large number of violations of the State law was heard and considered by the Board and instructions given to the Secretary to adjudicate them as far as possible.

The need of some more fixed source of income for the Board was freely discussed, and the plan of reregistration over the entire State annually was decided upon as the most promising plan for the sustenance of the Board.

W. HORACE HOSKINS, D.V.S.,
Secretary.

